# Comparison of three different surgical treatment methods in humeral shaft fractures

**DOI:** 10.11604/pamj.2022.42.88.34692

**Published:** 2022-06-02

**Authors:** Bekir Karagoz, Mustafa Erdem, Mustafa Cukurlu, Ismail Agir

**Affiliations:** 1 Adiyaman University Training and Research Hospital Department of Orthopaedics and Traumatology, Adiyaman, Turkey

**Keywords:** Humeral fracture, dual plate, intramedullary nail, locking plate

## Abstract

**Introduction:**

in this study, we aimed to compare the clinical and radiological results of single 4.5 mm locking compression plate (LCP), dual 3.5 mm LCP and intramedullary nailing (IMN) methods applied to the surgical treatment of humeral shaft fractures.

**Methods:**

the study included 77 patients (41 males, 36 females; mean age 46.38 years; range 18-74 years) with humeral shaft fractures treated with a single 4.5 mm LCP, dual 3.5 mm LCP and IMN between January 2016 and December 2020. Single 4.5 mm LCP (Group A) was applied to 31 (40.3%) patients, dual 3.5 mm LCP (Group B) to 20 (26%) patients and IMN (Group C) to 26 (33.8%) patients. The preoperative and postoperative data of the patients were analyzed from the hospital registry system. A short version of the disabilities of the arm, shoulder and hand (QuickDASH) questionnaire was used to evaluate functional outcomes.

**Results:**

as a result of the comparison of the rates of nonunion between the groups, a significantly lower rate of nonunion was observed in group B patients (p=0.027). While the rate of nonunion was 14% in the cases included in the study, no cases of nonunion were encountered in group B. There was no difference between the three groups in terms of demographic data and other postoperative complications.

**Conclusion:**

dual 3.5 mm LCP method is a suitable alternative to other surgical methods in the treatment of humeral shaft fractures, due to similar functional results and lesser nonunion.

## Introduction

Humerus shaft fractures are orthopedic injuries that can cause serious sequelae in patients. It constitutes 3-5% of fractures due to orthopedic trauma [1,2]. It occurs as a result of direct or indirect trauma, usually in sports-related activities or high-energy accidents. There are multiple treatment options in the treatment of humeral shaft fractures [3,4]. Because the humeral shaft is anatomically surrounded by dense muscles and vascularization is good, uncomplicated fractures are mostly treated conservatively [5]. The most common indications for surgical treatment are multiple trauma, vascular injury, segmental fractures and unsuccessful conservative treatment [2,6]. There are methods such as external fixation, single or dual plating, intramedullary nailing (IMN) and minimally invasive plating (MIPO) in surgical treatment. However, which method to choose is still a matter of debate. The method to be preferred has its own advantages and disadvantages. In open reduction and internal fixation, more rigid and anatomical reduction is achieved because the fracture fragments are manipulated more easily. However, in this method, there is a possibility of more detachment of the periosteum, more devitalization of tissues, and a higher rate of injury to the radial nerve [7]. The advantages of IMN include less damage to soft tissue, less stripping of the periosteum, which plays an important role in fracture union, and biomechanical load sharing of the implant [2,8,9]. Despite this, the rotator cuff may be injured due to insufficient exploration, and the fracture healing may be adversely affected by distraction at the fracture line during the placement of the nail [10-12]. In MIPO, the soft tissue is less damaged and the fracture biology is better preserved, but the radial and musculocutaneous nerve are at risk [13].

The number of studies comparing the effectiveness of the procedures applied in the surgical treatment of humeral shaft fractures is still insufficient in the literature. The number of studies evaluating the efficacy of fixation with dual plating is very few. The aim of this study is to compare the radiological and clinical results of fixation procedures with single 4.5 mm locking compression plate (LCP), dual 3.5 mm LCP and IMN used in the treatment of humeral shaft fractures.

## Methods

**Study design and setting:** in this retrospective study, patients who underwent surgery for humeral shaft fracture between January 2016 and December 2020 were determined by examining the hospital database. Fractures of the humeral shaft were defined as fractures of the humerus between the surgical neck region and the olecranon fossa [14].

**Study population:** in the examinations, 102 patients who were operated on for humeral shaft fractures were detected. Patients younger than 18 years of age (n=4), patients who were out of follow-up or with less than one-year of follow-up (n=11), tumour-related pathological fractures (n=5) and elbow or shoulder injuries (n=5) were excluded from the study. The remaining 77 patients were included in the study. 36 patients were female, 41 were male, and the mean age was 46.38±18.91 (18-74 years) years. Fractures occurred on the left side in 40 patients (51.9%) and on the dominant arm in 49 patients (62.9%). The mean follow-up period was 36.67 months (12-60 months). The patients were divided into three groups according to the surgical procedures: Group A consisted of 31 (40.3%) patients who underwent single 4.5 mm LCP, Group B consisted of 20 (26%) patients who underwent dual 3.5 mm LCP, and group C consisted of 26 (33.8%) patients who underwent IMN.

**Data resource and measurement:** by examining the hospital data system, data such as demographic characteristics of the patients, mechanism of injury, waiting time until surgery, duration of surgical operation, amount of intraoperative bleeding and postoperative complications (implant failure, nonunion, radial nerve injury, deep infection, limitation of movement in the shoulder and elbow) were recorded. The short version of the Disabilities of the Arm, Shoulder and Hand (QuickDASH) questionnaire was used to evaluate shoulder joint function [15]. The AO/Orthopedic Trauma Society classification of humeral shaft fractures was used to classify fracture types [16]. Union was evaluated using anteroposterior and lateral radiography at follow-ups. Union was defined as the absence of pain at the fracture line in the postoperative follow-up and the presence of callus formation in three cortices radiologically [17]. Failure of the fracture to heal in the first six months was considered as “nonunion” [18]. In addition, elbow and shoulder joint range of motion (ROM) was evaluated and recorded in the postoperative follow-up.

**Surgical procedure:** the operations were performed by three experienced surgeons. The choice of the surgical procedure to be applied to the patients was made according to the experience of the surgeon who will perform the procedure. The patients were evaluated by anesthesiologists before the operation, and general anesthesia or brachial plexus block was applied. Preoperative 75 mg/kg cefazolin sodium prophylaxis was applied to the patients. An arm tourniquet was not used during the operation.

**Figure 1:**
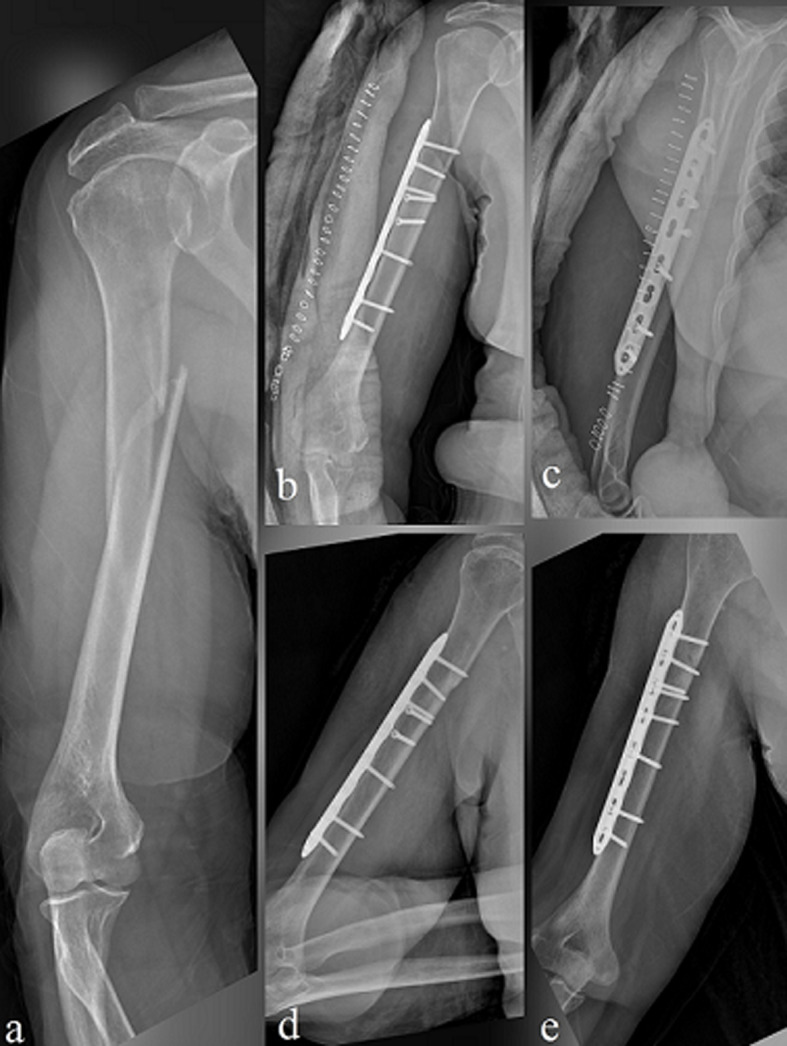
4.5 mm single LCP fixation; X-ray images of a 29-year-old male patient with a right humeral shaft fracture as a result of a sports injury: (a) first anteroposterior (AP) plane X-ray image; (b and c) early postoperative X-ray images; (d and e) X-ray images at the 6^th^-month postoperative control

**Fixation with a single 4.5 mm LCP:** the patient was placed in the supine position and the operated arm was placed on a hand table. An anterolateral incision was used and the radial nerve was carefully dissected according to the technique previously described [19]. The fracture was reduced with appropriate maneuvers and the reduction control was controlled by fluoroscopy. Interfragmentary screws were used when needed. Then, A single 4.5 mm LCP was placed on the anterolateral surface of the humerus using at least three screws on each side of the fracture. The radial nerve was preserved while the plate was placed. After the bleeding was controlled, a hemovac drain was placed. After the surgery, neurovascular examination was performed and recorded (Figure 1).

**Figure 2:**
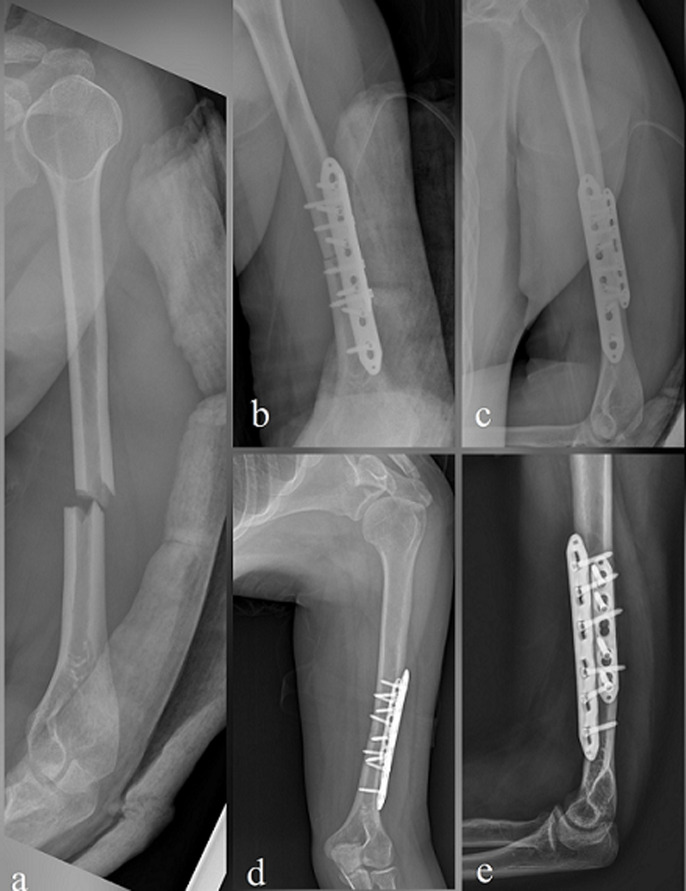
dual 3.5 mm LCP fixation; X-ray images of a 36-year-old woman with a left humeral shaft fracture as a result of a fall: (a) initial anteroposterior (AP) plane X-ray image; (b and c) early postoperative X-ray images; (d and e) X-ray images at 6^th^-month follow-up after surgery

**Fixation with dual 3.5mm LCP:** in the dual 3.5 mm LCP fixation, patients were placed supine position on the operating table and the operated arm was placed on the hand table. An anterolateral incision was used as in the single 4.5 mm LCP procedure. After the fracture was reduced, a 3.5 mm LCP was placed on the anteromedial surface of the humerus and at least two bicortical screws were sent to both side of the fracture. A 3.5 mm longer LCP was then placed on the anterolateral surface and stabilization was achieved by placing at least three bicortical screws on either side of the fracture. Similarly, bleeding was controlled and a hemovac drain was placed. Postoperative neurovascular examination was performed and recorded (Figure 2).

**Figure 3:**
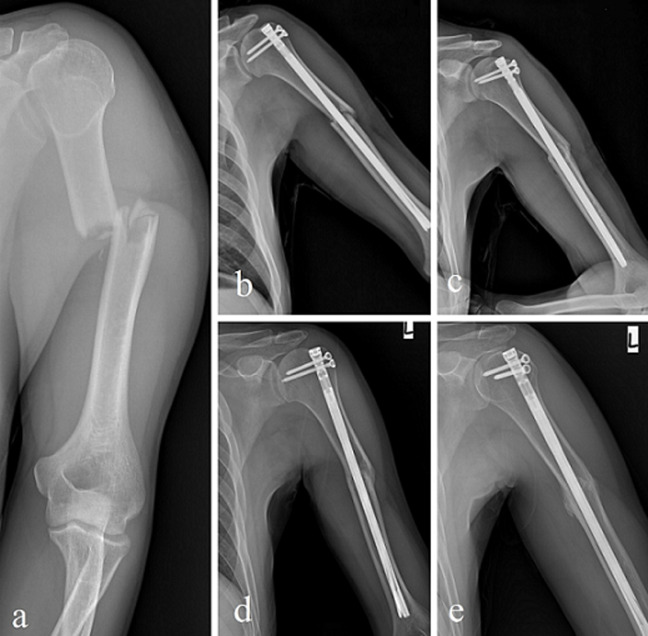
IMN fixation, X-ray images of a 43-year-old man with a left humeral shaft fracture as a result of a traffic accident: (a) initial anteroposterior (AP) plane X-ray image; (b and c) early postoperative X-ray images; (d and e) X-ray images at 6^th^-month follow-up after surgery

**Fixation with IMN:** after the patient was placed on the operating table in the sunbed position, a 4-5 cm incision was made on the skin and the deltoid cleft was separated. The deltoid muscle fibers and supraspinatus tendon were carefully separated and the nail insertion site was exposed. After closed reduction, the guide wire was sent up to the olecranon fossa under fluoroscopy control. In all patients, an InSafeLOCK® nail of appropriate size and diameter was placed after the medullary canal was reamed with a guide wire. In this nail, distal locking was achieved by inserting the endopin through the nail and placing it in the distal posterior cortex. Proximal locking screws were also placed using a guide. After control was achieved with scopy, the rotator cuff was repaired and the operation was finished. Postoperative neurovascular examination was performed and recorded (Figure 3).

**Postoperative follow-up:** active and passive elbow-shoulder movements were started from the second postoperative day. The patients were called for controls every four weeks for the first three months, and then once a month. Shoulder and elbow movements of the patients were evaluated at each control. At the end of one month, patients with limited range of motion in the shoulder or elbow joint were referred to the physical therapy and rehabilitation center.

**Statistical analysis:** IBM SPSS 23.0 software (IBM Corp., Armonk, NY, USA) was used for statistical analysis. While evaluating the data, descriptive statistical methods were used and the data were summarized. Shapiro-Wilk test was used for normality tests of continuous variables and Pearson Chi-square independence tests were used for independence tests between two categorical variables. Mann Whitney U Test and Kruskal-Wallis Test were used to compare data that did not show normal distribution. Relationships between classified variables were investigated with Chi-square tests of independence for 2x2 crosstabs. A p value of <0.05 was considered statistically significant.

**Ethical consideration:** this study was conducted after obtaining the necessary permission from Adiyaman University Non-Interventional Research Ethics Committee (Ethics committee approval number: 2021/09-18 and date: 16/11/2021). Data were collected anonymously from medical records. The patients who were contacted by phone received a positive opinion after the explanation about the study.

## Results

Descriptive data of 77 patients included in the study are summarized in Table 1. The most common trauma was falling (61%), and the fracture type was type A fracture (54.5%) according to the AO classification. The obtained data were analyzed by dividing into three groups and shown in Table 2. In comparisons between the groups, there was no statistically significant difference in terms of age, gender, fractured side, dominant side, body mass index, ASA (American Society of Anesthesiology) score, trauma type, fracture type, follow-up period and smoking (Table 2).

**Table 1:** demographic data of the patients

		N	%
**Gender**	Female	36	46.8
	Male	41	53.2
**Fracture side**	Left	40	51.9
	Right	37	48.1
**Dominant Side**	Left	2	2.6
	Right	75	97.4
**ASA Score**	1	28	36.4
	2	28	36.4
	3	16	20.8
	4	5	6.5
**Trauma type**	Fall	51	66.2
	Traffic accident	18	23.4
	Sports Injury	8	10.4
**Fracture type**	A	42	54.5
	B	29	37.7
	C	6	7.8
**Smoking**	+	42	54.5
	-	35	45.5
Total		77	100.0

* ASA: American Society of Anesthesiology

**Table 2:** comparison of demographic data by groups

Variables	SURGERY IMPLANTS			
	Group A (Single 4.5 mm LCP) (n=31)	Group B (Dual 3.5 mm LCP) (n=20)	Group C (IMN) (n=26)	p value
**Age**	44.61±19.10	47.55±17.97	47.58±19.95	0.814*
**Gender (Female:Male)**	14: 17	8: 12	14: 12	0.636**
**Dominant Side (Right:Left)**	30: 1	20: 0	25: 1	0.69**
**Fracture Side (Right:Left)**	12: 19	10: 10	15: 11	0.353**
**Body Mass İndex**	26.08±3.05	27.93±6.68	24.09±5.54	0.237*
**ASA Score (1:2:3:4)**	11:11:8:1	7:7:4:2	10:10:4:2	0.938**
**Trauma Type**				
**Fall**	21	14	16	
**Traffic accident**	6	4	8	0.838**
**Sports Injury**	4	2	2	
**Fracture Type**				
**A**	14	11	17	
**B**	14	7	8	0.613**
**C**	3	2	1	
**Follow-up (Month)**	52.87±24.02	44.25±22.88	40.38±26.93	0.129*
**Smoking (No:Yes)**	16:15	10:10	16:10	0.675**
**QuickDASH Score**	23.98±18.24	19.11±14.14	18.18±17.94	0.585*

* Kruskal Wallis Test, ** Chi-Square Test of Independence, LCP: Locking Compression Plate, IMN:Intrameduller Nailling, ASA: American Society of Anesthesiology

In the analysis of the QuickDASH score according to the three groups, the lowest score was found in Group C patients. However, in the statistical analysis performed between the groups, it was determined that the mean scores did not differ significantly according to the implant type (Table 2). In Table 3, waiting time until surgery, the duration of surgery and the amount of intraoperative bleeding were compared between the groups. According to these analyses, no significant difference was found between the groups in terms of time to surgery. In group C patients, the operative time and intraoperative blood loss were found to be significantly shorter than the other groups (p=0.003, p=0.000 respectively). When only group A and group B were compared, intraoperative blood loss was significantly higher in Group A patients (p=0.000).

**Table 3:** comparison of time to surgery, waiting time until surgery and amount of intraoperative bleeding between groups

Variables	SURGERY IMPLANTS			
	Group A (Single 4.5 mm LCP) (n=31)	Group B (Dual 3.5 mm LCP) (n=20)	Group C (IMN) (n=26)	p value
**Waiting time until surgery (day)**	3.71±3.20	3.65±2.28	4.42±2.40	0.223*
**Surgical time (minute)**	82.90±5.43	90.05±7.71	38.31±5.05	0.003**
**Amount of intraoperative bleeding (ml)**	214.68±22.32	165.50±10.63	109.81±12.53	0.000*/0.000**

The most common complication after surgery is nonunion. In the statistical analysis, the rates of nonunion between the groups were compared and it was observed that the rates of nonunion were significantly lower in group B patients (p=0.027). Nonunion was detected in 11 (14%) patients, and it was most common in group A with 8 (26%) patients, while no nonunion was observed in group B patients. Wound infection was not observed in any of the patients. In the analysis performed in terms of other postoperative complications, no significant difference was found between the groups (Table 4).

**Table 4:** comparison of postoperative complications between groups

Variables	Total (n=77)	SURGERY IMPLANTS			p value
		Group A (Single 4.5 mm LCP) (n=31)	Group B (Dual 3.5 mm LCP) (n=20)	Group C (IMN) (n=26)	
**Implant Failure**	%7.8 (6/77)	%12.9 (4/31)	%5 (1/20)	%3.8 (1/26)	0.385*
**Nonunion**	%14 (11/77)	%26 (8/31)	%0 (0/20)	%11.5 (3/26)	0.027*
**Radial Nerve Paralysis**	%5,2 (4/77)	%9.7 (3/31)	%5 (1/20)	%0 (0/26)	0.260*
**Wound Infection**	%0 (0/77)	%0 (0/31)	%0 (0/20)	%0 (0/26)	-
**Shoulder ROM Restriction**	%13 (10/77)	%9.7 (3/31)	%5 (1/20)	%23.1 (6/26)	0.290*
**Elbow ROM Restriction**	%9.1 (7/77)	%16.1 (5/31)	%10 (2/20)	%0 (0/26)	0.107*

* Chi-Square Test of Independence, LCP: Locking Compression Plate, IMN:Intrameduller Nailling, ROM: Range of Motion

## Discussion

The most important result obtained in this study is the lower rate of nonunion in dual 3.5 mm LCP application compared to single 4.5 mm LCP and IMN. Another important result is a higher incidence of shoulder joint range of motion limitation in patients who underwent IMN, despite a shorter operation time and less intraoperative bleeding. There are many studies in the literature on the surgical treatment of humeral shaft fractures [4,7,8]. In these studies, comparisons were made between plate and IMN, plate types or IMN types. However, as in our study, there is no study comparing single 4.5 mm LCP, dual 3.5 mm LCP and IMN together. There are few studies comparing single and dual plating [3,20]. Watt *et al*. [21] explained that dual plating is better than single plating due to better mechanical properties. In a biomechanical study by Kosmopoulos *et al*. [20] they reported that the torsional stiffness, posterior-anterior bending and lateral medial bending strengths of small dual 3.5 mm plate fixation were higher than the use of a single 4.5 mm long plate. In addition, there are many studies comparing plating and IMN fixation [4,5,7,9,22]. In a prospective study comparing IMN and plate fixation, they found higher shoulder pain and limitation of motion in the IMN group and higher elbow limitation in the plate group. However, union rates were found to be similar [22]. Jia *et al*. [4] in their meta-analysis study to compare IMN and plating technique, found that both methods were effective, but the rate of shoulder joint range of motion limitation was significantly higher in IMN. In our study, clinically similar results were obtained in the postoperative follow-up of all three procedures used in the surgical treatment of humeral shaft fractures. For this reason, it is thought that applying the procedure in which the surgeon is more experienced in implant selection and paying attention to the demographic characteristics of the patient while making the selection are important factors in increasing the success rates.

In the surgical treatment of humeral shaft fractures, postoperative complications that can seriously affect the quality of life of patients can be seen. One of the most important of these possible complications is nonunion. Nonunion may occur in 0-20% after conservative treatment of humeral shaft fractures and 3-25% after surgical treatment [6,23-25]. Some risk factors that may cause nonunion are as follows: open fractures, segmental fractures, comminuted fractures, smoking, diabetes, drugs such as nonsteroidal anti-inflammatory drugs, malnutrition and infection [11]. Different implants used in surgical treatment may cause different results. In a prospective study of 30 patients treated with LCP, the main postoperative complaint was pain, and the reported complication rate was 10% [26]. In many studies, nonunion after IMN was found to be between 0-30% and insufficient distal locking, insufficient fracture compression and infection were shown as causes [4,7-9]. Dual plating with a 3.5 mm LCP results in less soft tissue dissection and less periosteal scraping as a result of using a shorter incision than coating with a single 4.5 mm LCP, but requires additional medial dissection to place the plate on the anteromedial side [3]. However, in the study of Seo *et al*. [3] in which single 4.5 mm LCP and dual 3.5 mm LCP application were compared, no difference was found between the two groups in union at the 3^rd^ month after surgery, despite additional anteromedial soft tissue dissection. The union rates of the implant types used in our study were compared, and a statistically lower rate of nonunion was observed in those who underwent dual 3.5 mm LCP. No cases of nonunion were encountered in patients who underwent dual 3.5 mm LCP. In addition, this rate increased to 24% in cases where single 4.5 mm LCP was used. In the dual 3.5 mm LCP application, more rigid fixation was applied compared to the other two groups. In addition, the use of shorter plates and less damage to the periosteum compared to a single 4.5 mm LCP application are thought to be important factors in obtaining these results.

Radial nerve injury in humeral shaft fractures is the most common nerve injury after long bone fracture [27]. In a meta-analysis study involving 1045 patients, the rate of radial nerve injury accompanying humeral shaft fracture was found to be 12% [27]. There is a possibility of stretching, crushing and cutting the radial nerve during surgery. Postoperative radial nerve palsy is usually temporary, but it can be permanent in 2-3% of patients [11]. In our study, radial nerve palsy was detected with a rate of 5.2%. The highest rate of postoperative radial nerve palsy was seen in patients who underwent a single 4.5 mm LCP, but not in any of the patients who underwent IMN. All patients with radial nerve palsy recovered without the need for additional intervention. We think that the reason for the high rate of radial nerve palsy in patients using a single 4.5 mm LCP is extensive soft tissue dissection. We believe that careful soft tissue dissection and careful attention to the integrity of the nerve after the radial nerve is explored are very important in preventing this complication.

Other factors emphasized in the studies on the surgical treatment of humeral shaft fractures are the duration of the operation and the amount of intraoperative bleeding. In the study of Yuan *et al*. [14] in which MIPO and IMN were compared, it was determined that there was no difference in terms of amount of intraoperative bleeding and operation time. Wang *et al*. [8] found that the duration of surgery and the amount of intraoperative bleeding were significantly lower in patients who underwent IMN compared to the patients who underwent plate. In our study, shorter operative time and lower intraoperative bleeding were detected in patients who underwent IMN. Shorter incision, less soft tissue dissection, and preservation of fracture hematoma are presumed to be important factors in achieving these results. The limitations of this study are its retrospective nature, low number of patients and not long follow-up. In addition, the number of proximal locking screws in IMNs and the number of screws used in plate application are not standardized, which is one of the limitations of the study. Contribution to the literature can be made with studies that include more patients, have a long follow-up period, and are designed as randomized prospective.

## Conclusion

Dual 3.5 mm LCP application, which is used in the surgical treatment of humeral shaft fractures, has a high union rate and successful clinical results. The fact that the use of dual 3.5 mm plate allows a more rigid fixation compared to the use of a single 4.5 mm plate and IMN shows that this surgical method can be safely preferred.

### What is known about this topic

- While humeral shaft fractures are mostly treated conservatively, methods such as external fixation, single or double plating, intramedullary nailing and minimally invasive plating are used when surgical treatment is required;
- In the surgical treatment of humeral shaft fractures, postoperative complications that can seriously affect the quality of life of patients can be seen.

### What this study adds

- Dual 3.5 mm LCP application, which is used in the surgical treatment of humeral shaft fractures, has a high union rate and successful clinical results;
- Another important result is shorter operation time and less intraoperative bleeding in patients who underwent IMN in the surgical treatment of humeral shaft fractures.
